# Determinants of microbial community structure in supraglacial pool sediments of monsoonal Tibetan Plateau

**DOI:** 10.1128/spectrum.00754-24

**Published:** 2024-07-30

**Authors:** Heather Fair, Trinity L. Hamilton, Peter C. Smiley, Qiao Liu

**Affiliations:** 1 Department of Plant and Microbial Biology, University of Minnesota, Saint Paul, Minnesota, USA; 2 Soil Drainage Research Unit, Agricultural Research Service, USDA, Columbus, Ohio, USA; 3 Institute of Mountain Hazards and Environment, Chinese Academy of Sciences, Chengdu, China; 4 the Biotechnology Institute, University of Minnesota, Saint Paul, Minnesota, USA; University of Mississippi, University, Mississippi, USA

**Keywords:** cryoconite holes, supraglacial pools, monsoonal, Tibet, microbial communities, Chironomidae, habitat

## Abstract

Supraglacial pools are prevalent on debris-covered mountain glaciers, yet only limited information is available on the microbial communities within these habitats. Our research questions for this preliminary study were: (1) What microbes occur in supraglacial pool sediments of monsoonal Tibet?; (2) Which abiotic and biotic habitat variables have the most influence on the microbial community structure?; and (3) Does microbial composition of supraglacial pool sediments differ from that of glacial-melt stream pool sediments? We collected microbial samples for 16S rRNA sequencing and invertebrates for enumeration and identification and measured 14 abiotic variables from 46 supraglacial pools and nine glacial-melt stream pools in 2018 and 2019. Generalized linear model analyses, small sample Akaike information criterion, and variable importance scores were used to identify the best predictor variables of microbial community structure. Multi-response permutation procedure (MRPP) was used to compare taxa composition between supraglacial pools and stream pools. The most abundant phyla in supraglacial pool sediments were Proteobacteria, Actinobacteria, Bacteroidota, Chloroflexi, and Cyanobacteria. Genera richness, indicator genera richness, and *Polaromonas* relative abundance were best predicted by Chironomidae larvae abundance. *Angustibacter* and *Oryzihumus* relative abundance were best predicted by pH, *Acidiphilium* relative abundance was best predicted by turbidity, and *Sphingomonas* relative abundance was best predicted by glacier zone. Taxa composition was similar between supraglacial and stream pools at the class, genus, and ASV taxonomic levels. Our results indicate that Chironomidae larvae may play a keystone species role in shaping bacterial communities of supraglacial pools on debris-covered glaciers.

**IMPORTANCE:**

Glacier meltwater habitats (cryoconite holes, supraglacial pools, supraglacial ponds and lakes, glacial streams) and their biota have not been well-studied, especially on debris-covered glaciers in temperate monsoonal regions. Our study is the first to document the microbial community-habitat relationships in supraglacial pools on a debris-covered glacier in Tibet. Microbial genera richness, indicator genera richness, and *Polaromonas* relative abundance declined with increasing larval Chironomidae abundance, which is a novel finding that highlights the importance of larval insects in structuring microbial communities in supraglacial pools.

## INTRODUCTION

Mountain valley glaciers are slow-flowing frozen water masses with ephemeral lentic and lotic meltwater habitats in the supraglacial, englacial, and subglacial environments. Lentic glacier aquatic habitats can be viewed in a size-gradient ranging from the smallest cryoconite holes to the largest supraglacial lakes with supraglacial pools and ponds being intermediate in size. The microbial ecology and biogeochemistry of cryoconite holes of debris-free glaciers have been well-studied in the Arctic and Antarctic (1–4). Cryoconite granules occur on the accumulation and ablation zones of debris-free glaciers and ice fields or on the accumulation zone of debris-covered glaciers at high altitudes (5–7). Cryoconite forms through the accumulation of biological material of allochthonous and autochthonous origin around fine till that has been transported onto glacier surfaces (8). Cryoconite holes with meltwater develop at locations where cryoconite granules settle on the ice or snow and the albedo difference of the cryoconite and ice causes melting to occur. Microbes form biological aggregates of flocculent material within cryoconite holes because of the creation of a lentic environment within the weathering crust (9).

Differences in the relative abundance of microbial phyla have been observed between cryoconite holes within different geographic regions, such as Antarctica and the Arctic (10). Surface debris has been compared with surface meltwater where differences in microbial abundance were found (11), and differences in phyla abundance of glacial meltwater habitats were found between the Tibetan Plateau and other regions (12). As well, supraglacial ice surfaces of southern polythermal glaciers of the Tibetan Plateau had greater bacteria genera richness (71 to 89 genera) than the supraglacial ice surfaces of Tibetan Plateau glaciers influenced by the continental climate and westerlies air flows (40 to 57 genera) (13).

Even with a basic understanding of cryoconite hole microbial community structure, it is important to further understand the relationships between microbial communities and glacial habitat variables. Sediment texture, organic content, and pH were found to be predictors of Cyanobacteria and microalgae abundance, biovolume, and community structure in glacier habitats of supraglacial kames, cryoconite, and moraine in Svalbard, Arctic (14). pH was found to be an important habitat variable in structuring cryoconite hole microbial communities in the Karakorum Mountain range and in multiple regions around the world (15, 16). Increases in Cl- due to brine ice melting at the beginning of snowmelt results in increases in bacterial activity within entombed cryoconite holes in Antarctica (17). Primary productivity was found to be important in structuring microbial taxa composition in Antarctica (18).

Most microbial studies of debris-covered glaciers have focused on the debris communities rather than sediments or water in meltwater habitats (19, 20). Supraglacial pools on a debris-covered glacier in Tibet have on average mean surface area 2.1 to 5.5 times greater than cryoconite hole measurements reported from Svalbard, Canada, and Antarctica (1, 2, 17, 21–24). These pools also exhibited greater variation in water depths (e.g., 2 cm to >2 m deep), greater diversity in inorganic substrate richness with size classes up to large boulders, greater range of incision, and a greater range of water temperatures (24). Moreover, the regional climate may contribute to the formation of supraglacial pools, with the ice temperature, rainfall, and rainfall-induced debris flows all contributing to the development of the physical characteristics of pools.

**Fig 1:**
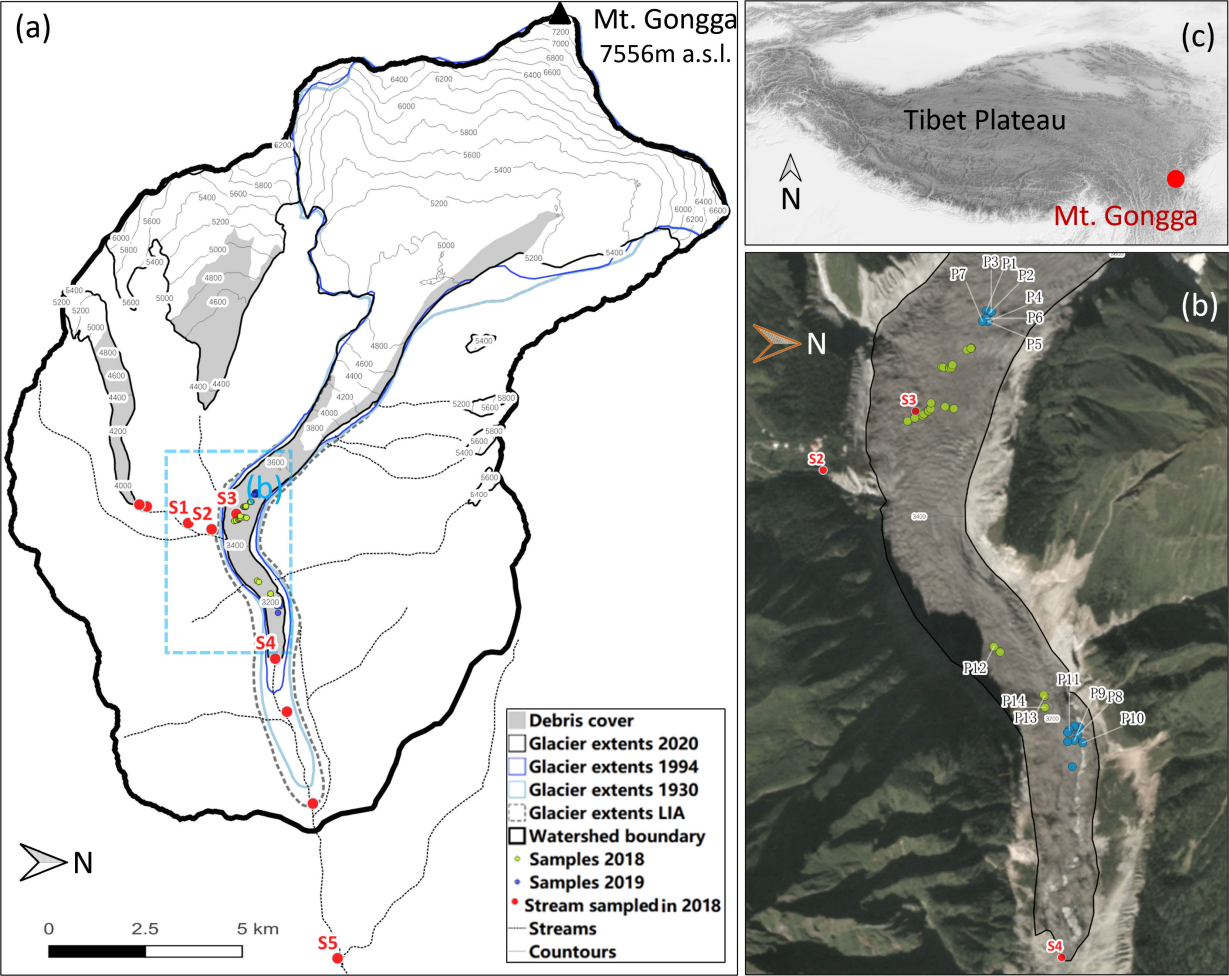
The Hailuogou Glacier catchment showing Glacier number 3 stream (smaller glacier on left) (a) and Hailuogou Glacier (a) and (b). The green dots in subfigure b depict the locations of supraglacial pools sampled in 2018 and the blue dots depict supraglacial pools sampled in 2019. Additionally, stream pools sampled are depicted with red dots in subfigures (a) and (b). Supraglacial pools and stream pools having enough DNA for microbial community analysis are labeled with combinations of letters and numbers. Subfigure (c) depicts the location of the Hailuogou Glacier catchment on Mt. Gongga in the eastern edge of the Tibetan Plateau [Glacier extents from (25)].

To our knowledge no information is available on bacterial communities within supraglacial pools on debris-covered glaciers in Tibet and only limited information is available on the bacterial communities within supraglacial ponds (12). Our objectives were to document the microbial taxa composition within the sediments of supraglacial pools on a debris-covered glacier in Tibet, to identify the best predictors of microbial communities in these pools, and to compare microbial taxa composition between supraglacial pools and stream pools within periglacial streams. Our research questions were: (1) What microbes occur within the sediments of supraglacial pools on a debris-covered glacier?; (2) Which habitat (physical, chemical, biotic) variables have the most influence on the microbial community structure in supraglacial pools?; and (3) How does microbial taxa composition of supraglacial pools compare with pools in periglacial, supraglacial, and proglacial streams? To address our research questions, we examined bacterial communities from supraglacial pool sediments and evaluated the relationships of microbial community structure with physical variables, chemical variables, and invertebrates within supraglacial pools during the summer ablation seasons of 2018 and 2019 (Fig. 1). We also compared the microbial taxa composition between the supraglacial pools and glacial-melt stream pools.

## MATERIALS AND METHODS

### Study area

Mt. Gongga is in the southeastern temperate monsoonal region of the Tibetan Plateau and has 74 glaciers classified as debris-covered, which is characteristic of high mountain valley glaciers in this region. The Hailuogou Glacier is approximately 13 km in length and 24.7 km^2^ in area, with an accumulation zone originating at 7,556 m a.s.l., and its ablation zone terminating at 2,990 m a.s.l. on the eastern slope of Mt. Gongga (Fig. 1). The Hailuogou Glacier has a 1,080 m icefall, which delineates the accumulation and ablation zones and provides a consistent supply of ice avalanche material to the ablation zone. The northern edge of the glacier close to the icefall has experienced significant valley wall deformation recently (25). The upper section of the ablation zone is covered by a thin layer of debris (< 3 cm), and the lower part is covered by a thicker debris layer that reaches up to 1.5 m thick (26).

Meltwater-filled supraglacial pools on the Hailuogou Glacier ablation zone and periglacial, proglacial, and supraglacial stream pools were examined for physical and chemical environmental variables and microbial community characteristics. During June 2018, we sampled 21 supraglacial pools in the upper ablation zone and seven pools on the lower ablation zone. We also sampled: (1) four stream pools above the Hailuogou Glacier in the Glacier #3 proglacial stream; (2) four stream pools on the Hailuogou mainstem; and (3) one stream pool from a supraglacial stream. In August 2019, we sampled nine supraglacial pools in the upper ablation zone and nine supraglacial pools on the lower ablation zone. In all, we sampled 46 supraglacial pools and nine stream pools. We selected pools as we encountered them during our search efforts and then searched for surrounding pools in a 20 m circular pattern before continuing to search up or down glacier. The accessibility of the terrain affected our ability to locate supraglacial pools especially on the lower ablation zone with thicker debris and crevasse-rich terrain. In 2019, accessing the lower ablation zone was difficult because a landslide damaged the staircase that provided access to the glacier. We ensured that we did not repeatedly sample any pools by comparing the GPS coordinates of pools sampled in 2018 and 2019.

### Microbial community collection and characterization

Using aseptic techniques, we collected sediment samples from supraglacial and stream pools for microbial analyses prior to conducting physical measurements, chemical measurements, and invertebrate sampling. Approximately 5 g of sediment was collected with a spoon, water was decanted from the sediments, and the sediments were placed in a sterile tube and preserved in 15 mL of RNALater. Preserved sediments were later refrigerated and transported to the laboratory for DNA extraction. DNA was extracted using a PowerSoil DNA Isolation Kit (Qiagen, Inc., Germantown, Maryland). Bulk DNA concentrations were determined using a Qubit dsDNA HS Assay kit (Molecular Probes, Eugene, Oregon) and Qubit Fluorometer (Invitrogen, Carlsbad, California. DNA was submitted to the University of Minnesota Genomics Center for amplicon sequencing targeting the V4 region of the 16S rRNA gene. The University of Minnesota Genomics Center prepared dual indexed Nextera XT DNA libraries with their improved protocol that enables the detection of taxonomic groups that are often undetected (27) and sequenced the libraries using a 2 × 300 v3 flow cell (Illumina, San Diego, California). Demultiplexed samples with primers removed were used to conduct the DADA2 workflow (28). Paired-end raw reads were processed to denoise, detect, and remove chimeras, and taxonomy assigned using the SILVA database (v138, 29). Thirty-two supraglacial pools and five stream pools did not contain enough DNA for further analyses. 16S rRNA sequences from 14 supraglacial pools and five stream pools were comprised into objects with the phyloseq function (30). Before conducting statistical analyses singletons, unidentified ASV phyla, mitochondrial DNA, and chloroplast sequences were removed from the data set.

### Physical and chemical measurements and invertebrate sampling

The methods used for the measurement of all predictor variables within supraglacial pools were previously described (24). For our analysis, we selected a subset of physical and chemical predictor variables identified as potentially important predictors of community structure in supraglacial pools (24). The surface area, mean water depth, and mean ice to water surface depth were obtained for each supraglacial pool. Turbidity was visually estimated and classified as: (1) very clear – bottom substrate visible; (2) clear – bottom substrate visible but with slight haze in the water column; (3) turbid – water is a gray color and the bottom substrate visible, but difficult to see; or (4) very turbid – water color is gray or brown and bottom substrate is not visible. Water temperature and specific conductivity were measured with a multiparameter meter (YSI Professional Plus). Pool substrate composition was determined by selecting 100 random substrate pieces (i.e., clastic sedimentary rocks) from each supraglacial pool and using a gravelometer to classify each piece into one of 14 size classes (≤2, 2.8, 4, 5.6, 8, 11, 16, 22.6, 32, 45, 64, 90, 128, > 180 mm). Subsequently, we calculated mean grain size and substrate richness. Substrate richness is the number of different substrate size classes.

For each supraglacial pool we used GPS coordinates and ArcMap (31) to calculate the difference in distances of the pools from the northern and southern glacier boundaries (difNB1SB1) and the number of supraglacial pools within 5 m. These two glacier landscape variables described the relative location of the sampled supraglacial pools with respect to the glacier edge or the distance of sampled pools from a local source of microbial colonists on the glacier.

We collected invertebrates from supraglacial pools with a dip net (mesh size 750–800 µm). Invertebrates were preserved in 99% ethanol and identified in the laboratory using a dissecting microscope and taxonomic keys (32). The occurrence and abundance of Collembola, Chironomidae, Isotomidae, and invertebrates (Chironomidae and Isotomidae combined) within each supraglacial pool were calculated and used as predictor variables.

### Statistical analysis

For question number 1, to document the microbial taxa present and trends in microbial taxa relative abundance in the supraglacial pools, we used the 16S rRNA gene ASVs to document which taxa are present at the phyla and genus level and their relative abundances. The relative abundance of each taxa within a pool was calculated by dividing the total abundance of each ASV within each pool by the abundance of all ASVs in each pool. Likewise, to calculate the proportion of each taxa within all pools we summed the abundance of each ASV taxa across all pools and divided this sum by the abundance of all ASV taxa across all pools.

For question number 2, we identified the best predictors of microbial community structure within supraglacial pools using a combination of generalized linear model analyses, Akaike’s Information Criterion analyses, and variable importance scores. Microbial community response variables calculated based on the genus-level ASVs included (1) Hill genera richness, (2) *Angustibacter* relative abundance, (3) *Oryzihumus* relative abundance, (4) *Acidiphilium* relative abundance, (5) *Sphingomonas* relative abundance, (6) *Polaromonas* relative abundance, (7) Indicator genera relative abundance, (8) Hill indicator genera richness, (9) Principal Components Analysis (PCA) axis 1 site scores, and 10) PCA axis 2 site scores. Hill genera richness and indicator genera richness (q = 0) were calculated using the iNEXT function (iNEXT package, 33). *Angustibacter*, *Oryzihumus*, and *Acidiphilium* relative abundances were selected because these are the three most abundant genera of supraglacial pools. *Sphingomonas* and *Polaromonas* relative abundances were calculated because these are microbial genera documented to occur in polar regions or in glacier meltwater habitats (34). PCA axis 1 and 2 site scores served as indicators of trends in taxa composition occurring among the supraglacial pools. Prior to conducting PCA, rare microbial genera found in <10% of the 14 supraglacial pools were removed and the relative abundance data were chord transformed using the decostand function (vegan package, 35). PCA analyses were conducted with PC-ORD (36). Indicator genera for supraglacial pools (i.e., *Hymenobacter* and KD3-10) were identified using Indicator Species Analysis as described below and indicator genera relative abundance and richness describes the proportion and diversity of genera strongly associated with supraglacial pools.

Prior to conducting formal analysis, we first ran an initial generalized linear model analyses with the glm function (stats package, 37), the Gaussian error family, and a subset of predictor variables to determine if the model residuals met the assumptions of normality and homoscedasticity. The selected predictor variables were (1) specific conductivity, (2) mean grain size, (3) difNB1SB1, (4) number of pools within 5 m, and (5) invertebrate abundance. We assessed the normality of the model residuals with the shapiro.test function (stats package, 37). The homoscedasticity of the model residuals was evaluated by graphing the residuals and fitted values and assessing the distribution of points in the graphs.

Indicator genera relative abundance was the only response variable that met the assumptions of normality and homoscedasticity. Genus richness and indicator genera richness were log(x + 1) transformed prior to generalized linear model analysis. The relative abundances of *Angustibacter*, *Oryzihumus*, *Acidiphilium*, *Sphingomonas,* and *Polaromonas* were arcsine squareroot transformed prior to generalized linear model analysis. PCA axis 1 site scores were log (x + 11.19) and PCA axis 2 site scores were log (x + 13.51) prior to analysis. In the first stage of the analysis for each response variable we conducted generalized linear model analysis (glm function, Gaussian error family) with single variable models developed with 20 predictor variables to identify the top three predictor variables. The predictor variables were (1) elevation, (2) glacier zone (upper or lower ablation zone), (3) water temperature, (4) pH; (5) specific conductivity, (6) turbidity, (7) mean grain size, (8) substrate richness, (9) difNB1SB1, (10) number of pools within 5 m, (11) mean surface area, (12) mean water depth, (13) mean ice surface to water surface depth, (14) mean surface area to depth ratio, (15) Chironomidae abundance, (16) Isotomidae abundance, (17) invertebrate (Chironomidae and Isotomidae) abundance, (18) Chironomidae occurrence (presence/absence), (19) Isotomidae occurrence, and (20) invertebrate occurrence. The small sample Akaike Information Criterion (AICc) scores were used to identify the top five models (i.e., predictor variables). Single variable models with ΔAICc (the difference in AICc of a model and the minimum AICc) ≥5 were excluded from consideration. AICc and ΔAICc scores were calculated using the aictab function (AICcmodavg package, 38). We also conducted pairwise Pearson correlation tests (cor.test function, psych package, 39) among the predictor variables and eliminated predictor variables exhibiting moderate-to-strong multicollinearity (i.e., |r| > 0.50).

For the second stage of analysis, we developed single and multiple variable models for each response variable using the predictor variables identified in stage one analysis. For each response variable we also developed an intercept only model to serve as a null model. In the development of multiple variable models, we only developed models containing a maximum of three predictors to avoid issues associated with overfitting (40). We obtained the AICc score, ΔAICc, and the Akaike weight (Wi) using the aictab function. Three response variables (Genera richness, *Acidiphilium* relative abundance, indicator genera richness) resulted in the inclusion of only one predictor variable in stage 2 analyses. For these response variables, the focal predictor variable was considered the most important one if the AICc score for the one predictor variable model was <2 AICc units from the null model. We also obtained the model coefficients and calculated bootstrapped 95% confidence intervals (confint function, stats package, 37) with 1,000 iterations for the most important predictor variables. For the remaining seven response variables, we identified the most important predictor variable with the variable importance scores (importance function, AICcmodavg package). For these response variables, we obtained the model averaged coefficients and the 95% confidence intervals using the modavgShrink function (AICcmodavg package).

Our analyses for question number 3 consisted of a combination of ordination analysis and multi-response permutation procedure (MRPP) to determine if the microbial taxa composition at the class level, genus level, and ASV level differed between supraglacial pools and stream pools. Our ordination analyses followed the same process as described previously except with chord-transformed relative abundance of microbial classes, genera, and ASVs found in 14 supraglacial pools and five stream pools. Rare microbial classes, genera, and ASVs were removed prior to ordination and MRPP analyses. We used the Mantel test to compare PCA axis 1 and 2 site scores among the class, genera, and ASV PCAs to assess the similarity in our PCA results among the three taxonomic resolutions. We also conducted Indicator Species Analyses (41) to identify which classes, genera, and ASVs serve as indicator taxa for supraglacial pools and stream pools. PCA, MRPP, Mantel tests, and Indicator Species Analyses were conducted using PC-ORD (36).

## RESULTS

### Supraglacial pool microbial community characteristics

The 14 supraglacial pools contained 773,031 16S rRNA ASVs that included 46 bacteria phyla, five Archaea phyla, and 500 bacterial and archaeal genera. The five phyla with the greatest relative abundance of ASVs across all supraglacial pools were Proteobacteria (29%), Actinobacteria (19%), Bacteroidota (14%), Chloroflexi (8%), and Cyanobacteria (5%). The five most abundant genera found in supraglacial pools consisted of *Angustibacter* (4%), *Oryzihumus* (3%), *Acidophilous* (3%), *Sphingomonas* (2%), and *Polaromonas* (2%) (Table 1). Thirty-seven percent of ASVs were unclassified at the genus level.

**TABLE 1:** Number and percent of amplicon sequencing variants (ASVs) of the 13 most abundant microbial genera that compromised 31% of the total ASVs of bacteria within fourteen supraglacial pools on the debris-covered Hailuogou Glacier, Ganze Tibetan Autonomous Region, China, June 2018 and August 2019

Phylum	Genera	Number of ASVs	Percent of ASVs
Actinobacteriota	*Angustibacter*	28060	4
Actinobacteriota	*Oryzihumus*	25117	3
Proteobacteria	*Acidiphilium*	22475	3
Proteobacteria	*Sphingomonas*	19462	2
Proteobacteria	*Polaromonas*	17087	2
Proteobacteria	*1174–901-12*	16028	2
Bacteroidota	*Ferruginibacter*	15681	2
Bacteroidota	*Flavobacterium*	15637	2
Actinobacteriota	*Gaiella*	12651	2
Cyanobacteria	*Chamaesiphon PCC-7430*	12575	2
Proteobacteria	*Methylotenera*	11339	1
Actinobacteriota	*CL500-29 marine group*	11231	1
Firmicutes	*Bacillus*	10046	1

**Fig 2:**
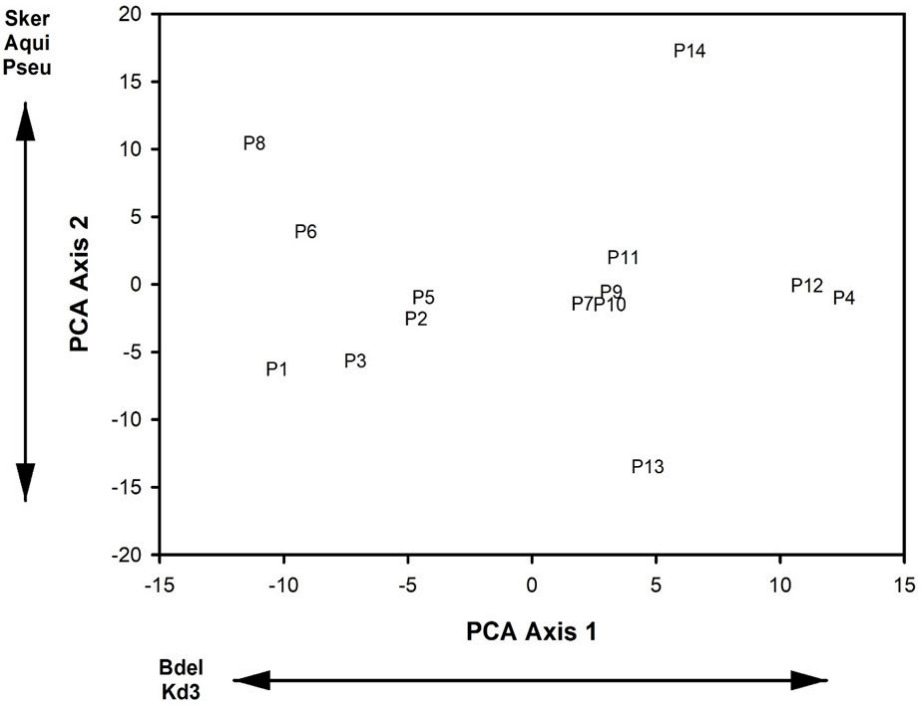
Site scores from the first two axes of the Principal Components Analysis (PCA) of the relative abundances of microbial genera in fourteen supraglacial pools on the Hailuogou Glacier, Ganze Tibetan Autonomous Region, China, June 2018, and August 2019. Microbial genera abbreviations are: Bdel – Bdellovibrio; Kd3 - KD3.10; Sker – Skermanella; Aqui – Aquipuribacter; and Pseu – Pseudorhodoplanes.

Mean genera richness was 75.39 (SE = 20.84) in the supraglacial pools. Mean indicator genera relative abundance was 0.006 (SE = 0.11) and mean indicator genera richness was 1.64 (SE = 2.06). The means and SE of the relative abundance of our focal microbial genera were (1) 3.6% (SE = 0.26%) – *Angustibacter* relative abundance, (2) 3.2% (SE = 0.21%) – *Oryzihumus* relative abundance, (3) 2.9% (SE = 0.15%) – *Acidiphilium* relative abundance, (4) 2.5% (SE = 0.21%) – *Sphingomonas* relative abundance, and (5) 2.0% (SE = 0.21%) - *Polaromonas* relative abundance. The first two PCA axes of the relative abundances of microbial genera in supraglacial pools accounted for 33.6% of the variance in the data set, and the calculated eigenvalues from both axes were greater than the broken stick eigenvalues. PCA axis 1 loadings indicated that decreasing site scores corresponded with increasing relative abundances of *Bdellovibrio* and *KD3.10* (Fig. 2). PCA axis 2 loadings indicated that increasing site scores corresponded to increases in relative abundances of *Skermanella, Aquipuribacter*, and *Pseudorhodoplanes* (Fig. 2).

### Physical, chemical, and biotic characteristics of supraglacial pools

Mean length and width of the 14 supraglacial pools containing sufficient microbial DNA for analyses was 57.8 cm (SE = 6.8), and the mean area was 3,552 cm^2^ (SE = 732). The mean water depth of the supraglacial pools was 17.4 cm (SE = 4.7), and the mean ice to water surface depth was 10.3 cm (SE = 2.8). The mean number of supraglacial pools within 5 m was 0.36 (SE = 0.13). Mean grain size within the pools was 16.2 mm (SE = 2.6), and the mean substrate richness was 10.5 (SE = 1.0). Water temperatures ranged from 0.1°C to 4.5°C with a mean of 1.2°C (SE = 0.4). Mean specific conductivity was 26.5 µS/cm (SE = 2.7) and the mean pH was 7.73 (SE = 0.27). Five of the 14 supraglacial pools contained larval Chironomidae, and two pools contained larval Isotomidae. None of the supraglacial pools contained both taxa in the same pool. Mean Chironomidae abundance in supraglacial pools was 1.31 (SE = 0.35), and mean Isotomidae abundance was 0.5 (SE = 0.13).

### Evaluation of the best predictors of microbial response variables

Stage 1 analyses identified between one and three variables for each response variable to be retained for stage 2 analyses (Table 2). The most frequently retained predictor variables were Chironomidae abundance and pH, and these were used in stage 2 analysis of four to five response variables. See Table S1 for a summary of the AICc scores for the top ten models from each response variable.

**TABLE 2:** Predictor variables identified in stage 1 analyses for use in stage 2 analyses to identify the models that best predict microbial response variables in supraglacial pools on the Hailuogou Glacier, Ganze Tibetan Autonomous Region, China, June 2018, and August 2019^*a*^

Response variable	Predictor variables used
Genera richness	Chironomidae larvae abundance
*Angustibacter* relative abundance	pH, Chironomidae larvae abundance, turbidity
*Oryzihumus* relative abundance	pH, Chironomidae larvae abundance, number of pools within 5 m
*Acidiphilium* relative abundance	turbidity
*Sphingomonas* relative abundance	Zone, Isotomidae abundance
*Polaromonas* relative abundance	Chironomidae larvae abundance, turbidity
Indicator genera relative abundance	Number of pools within 5 m, elevation, pH
Indicator genera richness	Chironomidae larvae abundance
PCA axis 1 site scores	Invertebrate occurrence, pH, specific conductivity
PCA axis 2 site scores	Substrate richness, difNB1SB1, invertebrate occurrence

^*a*^ Within each row predictor variables are arranged in order from least AICc (small sample Akaike’s Information Criterion) score to the greatest. Abbreviations are: PCA – Principal Components Analysis; difNB1SB1 - difference in distances of the pools from the northern and southern glacier boundaries.

Stage 2 AICc comparisons involving null models indicated that none of the measured predictor variables were able to predict indicator genera relative abundance, PCA axis 1 site scores, and PCA axis 2 site scores (Table 3). For the remaining seven variables, we found that the best models contained one to two predictor variables and were at least 2.96 AICc units less than their associated null models. See Table 3 for all results from the AICc comparisons for all ten microbial response variables.

**TABLE 3:** Summary of the number of parameters (k), difference in small sample Akaike information criterion (AICc) between each model and the model with the minimum AICc (ΔAICc), and the Akaike weight (Wi) from the five best models and the null model from stage 2 generalized linear model analyses of microbial Hill genera richness, *Angustibacter* relative abundance, *Oryzihumus* relative abundance, *Acidiphilium* relative abundance, *Sphingomonas* relative abundance, *Polaromonas* relative abundance, indicator genera relative abundance, indicator genera richness, Principal Components Analysis axis 1 site scores, and Principal Components Analysis axis 2 site scores^*a*^

Response variable	Model	K	△AICc	Wi
Genera richness	Chironomidae abundance	3	0.00	1.00
	Null	2	19.31	0.00
*Angustibacter* RA	Chironomidae abundance + pH	4	0.00	0.82
	Chironomidae abundance + pH + turbidity	5	3.65	0.13
	pH	3	7.69	0.02
	pH + turbidity	4	8.44	0.01
	Chironomidae abundance	3	8.99	0.01
	Null	2	12.59	0.00
*Oryzihumus* RA	Chironomidae abundance +pH	4	0.00	0.42
	pH	3	1.46	0.20
	Chironomidae abundance	3	2.98	0.10
	pH +number of pools within 5 m	4	3.84	0.06
	Number of pools within 5 m	4	3.86	0.06
	Null	2	4.02	0.06
*Acidiphilium* RA	Turbidity	3	0.00	0.99
	Null	2	8.68	0.01
*Sphingomonas* RA	Zone	3	0.00	0.68
	Null	2	2.96	0.15
	Zone + Isotomidae abundance	4	3.83	0.10
	Isotomidae abundance	3	4.60	0.07
*Polaromonas* RA	Chironomidae abundance +turbidity	4	0.00	0.75
	Chironomidae abundance	3	2.53	0.21
	Turbidity	3	6.23	0.03
	Null	2	10.64	0.00
Indicator genera RA	Number of pools within 5 m	3	0.00	0.27
	Null	2	0.35	0.23
	Elevation	3	0.95	0.17
	pH	3	1.18	0.15
	Number of pools within 5m + pH	4	2.94	0.06
	Number of pools within 5m + elevation	4	3.12	0.06
Indicator genera richness	Chironomidae abundance	3	0.00	0.95
	Null	2	6.07	0.05
PCA axis 1	Invertebrate occurrence	3	0.00	0.27
	Null	2	0.17	0.25
	pH	3	0.93	0.17
	Invertebrate occurrence +pH	4	1.39	0.14
	Specific conductivity	3	2.44	0.08
	pH +condspc	4	3.86	0.04
PCA axis 2	Null	2	0.00	0.33
	Substrate richness	3	0.71	0.23
	difNB1SB1	3	1.88	0.13
	Invertebrate occurrence	3	1.98	0.12
	Substrate richness +difNB1 SB1	4	2.72	0.08
	Substrate richness +invertebrate occurrence	4	3.68	0.05

^*a*^ We conducted microbial sampling and habitat measurements in supraglacial pools on the hailuogou glacier, Ganze Tibetan Autonomous Region, China, June 2018, and August 2019. Abbreviations are: RA - relative abundance; PCA – Principal Components Analysis; difNB1SB1 – difference in distance of pools from northern and southern glacier boundaries.

**Fig 3:**
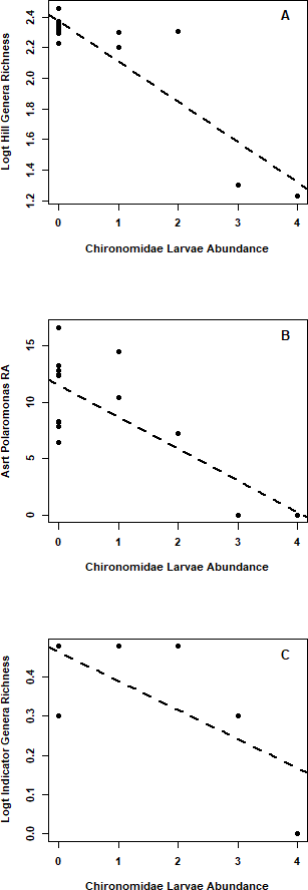
Predicted relationships between Chironomidae larvae abundance and (**A**) Hill Genera richness (log transformed), (**B**) Polaromonas relative abundance (RA) (arcsine transformed), and (**C**) Indicator species genera richness (log transformed) in supraglacial pools on the Hailuogou Glacier, Ganze Tibetan Autonomous Region, China, June 2018, and August 2019.

**Fig 4:**
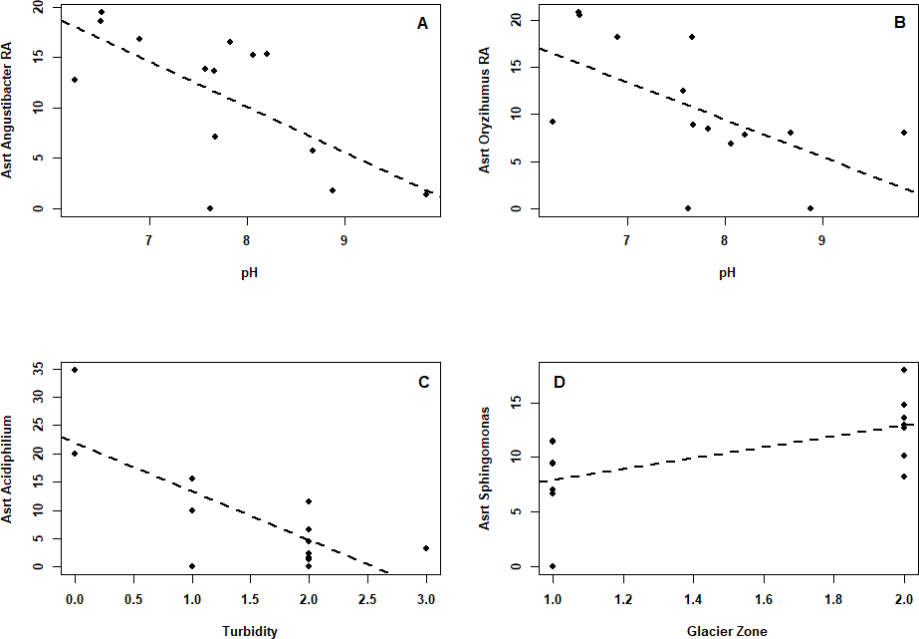
Predicted relationships between pH and (A) Angustibacter relative abundance (RA) (arcsine square root transformed), and (B) Oryzihumus RA (arcsine squareroot transformed), turbidity and (C) Acidiphilium RA (arcsine squareroot transformed), and glacier zone (1 = upper and 2 = lower) and (D) Sphingomonas RA (arcsine squareroot transformed), in supraglacial pools on the Hailuogou Glacier, Ganze Tibetan Autonomous Region, China, June 2018 and August 2019.

Null model comparisons and importance values indicated that the Chironomidae abundance was the best predictor of genera richness, *Polaromonas* relative abundance, and indicator genera richness (Fig. 3; Tables 3 and 4). Importance values also indicated that *Angustibacter* relative abundance and *Oryzihumus* relative abundance were best predicted by pH, *Acidiphilium* relative abundance was best predicted by turbidity, and *Sphingomonas* relative abundance was best predicted by zone (Fig. 4; Table 4). The 95% confidence intervals for all of the best predictors for these seven response variables did not overlap 0. Genera richness, *Polaromonas* relative abundance, and indicator genera richness decreased with increasing Chironomidae abundance (Fig. 3). *Angustibacter* relative abundance and *Oryzihumus* relative abundance decreased with increasing pH (Fig. 4). *Acidiphilium* relative abundance increased with decreasing turbidity and *Sphingomonas* relative abundance was greater in the lower glacier zone (Fig. 4).

**TABLE 4:** Summary of importance values and the generalized linear model analysis coefficient estimates and 95% CI (95% CI) for the predictor variables used in stage two analysis to identify the best predictor variable of microbial genera richness, *Angustibacter* relative abundance, *Oryzihumus* relative abundance, *Acidiphilium* relative abundance, *Sphingomonas* relative abundance, *Polaromonas* relative abundance, indicator genera relative abundance, indicator genera richness, Principal Components Analysis axis 1 site scores, and Principal Components Analysis axis 2 site scores in supraglacial pools on the Hailuogou Glacier, Ganze Tibetan Autonomous Region, China, June 2018 and August 2019^*c*^

Response variable	Predictor variable	IV	CE	95%
Genera richness	Chironomidae abundance	^*a*^	**−0.26^*a*^**	−0.34–−0.19^*a*^
*Angustibacter* RA	pH	0.98	**−3.96**	−6.29–−1.63
*Angustibacter* RA	Chironomidae abundance	0.96	**−2.75**	−4.59–−0.92
*Angustibacter* RA	Turbidity	0.16	0.26	−1.46–1.98
*Oryzihumus* RA	pH	^*a*^	**−3.95^*a*^**	−7.05–−0.85^*a*^
*Acidiphilium* RA	Turbidity	^*a*^	**0.06^*a*^**	−12.79–−4.43^*a*^
*Sphingomonas* RA	Zone	^*a*^	4.98	1.23–8.74
*Polaromonas* RA	Chironomidae abundance	0.96	**−2.27**	−3.88–−0.65
*Polaromonas* RA	Turbidity	0.78	2.04	−0.72–4.80
Indicator genera RA	Number of pools within 5 m	^*b*^	^*b*^	^*b*^
Indicator genera richness	Chironomidae abundance	^*a*^	**−0.07**	−0.12–−0.03
PCA Axis 1	Invertebrate occurrence	^*b*^	^*b*^	^*b*^
PCA Axis 2	Substrate richness	^*b*^	^*b*^	^*b*^

^*a*^ Importance values not calculated because stage two analysis only involved one predictor variable or only one predictor variable was better than the null model. The model coefficient reported is from the best model and the 95% CI was calculated with the confint function instead of modavgshrink function.

^*b*^ Importance value, model averaged coefficient and 95% confidence intervals not calculated because best model was within 1 AICc unit of the null model.

^*c*^ Bolded coefficient estimates are those with 95% confidence intervals that do not overlap 0. Abbreviations are: IV – importance value; CE – coefficient estimate; RA - relative abundance; PCA – Principal Components Analysis.

### Comparison of microbial taxa composition of supraglacial pools and stream pools

**Fig 5:**
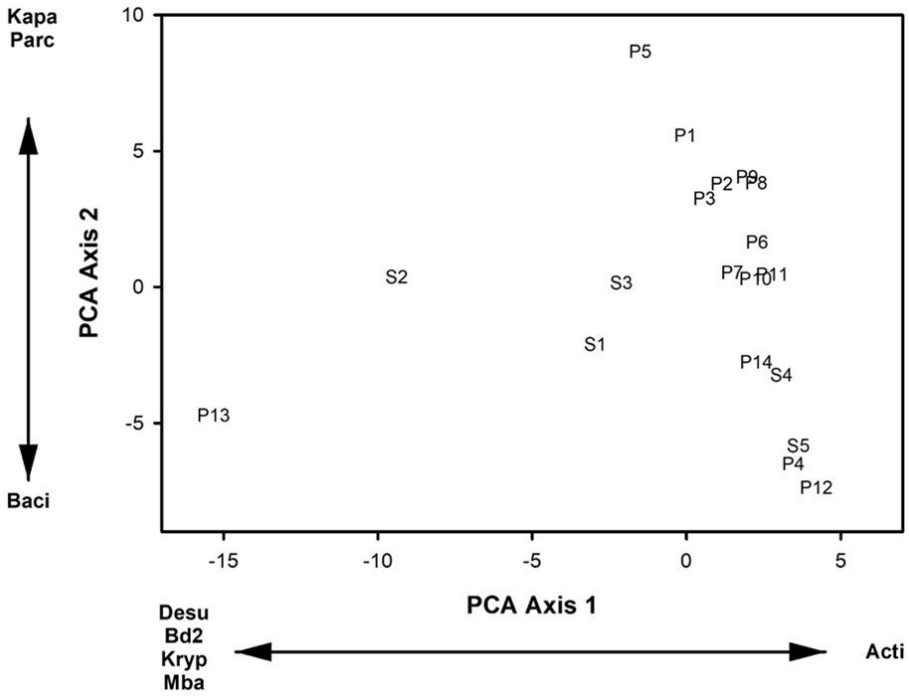
Site scores from the first two axes of the Principal Components Analysis of the relative abundances of microbial classes in 14 supraglacial pools and five stream pools on the Hailuogou Glacier, Ganze Tibetan Autonomous Region, China, June 2018, and August 2019. Microbial class abbreviations are: Acti - Actinobacteria; Desu - Desulfobulbia; Bd2 – BD2-11 terrestrial group; Kryp – Kryptonia; Mba – MB-A2-108; Kapa – Kapabacteria; Parc - Parcubacteria; Baci – Bacilli. Supraglacial pool sites are those letter number combinations that begin with P and stream pool sites are those letter number combinations that begin with S.

Four bacterial classes common across all supraglacial pools and stream pools were Actinobacteria, Alphaproteobacteria, Bacteroidia, and Gammaproteobacteria. Indicator Species Analysis identified vadinHA49 [IV (indicator value) =70.2, *P* = 0.030] and Deinococci (IV = 69.6, *P* = 0.048) as indicator classes of supraglacial pools and Rhodothermi (IV = 57.3, *P* = 0.047), Campylobacteria (IV = 48.3, *P* = 0.028), and Kryptonia (IV = 37.6, *P* = 0.036) as indicator classes of stream pools. The first two axes from the class PCA with supraglacial pools and stream pools accounted for 45.0% of the variance in the data and the eigenvalues from both axes were greater than the broken stick eigenvalues. PCA axis 1 loadings indicated that increasing site scores corresponded with increasing relative abundances of Actinobacteria and decreasing relative abundances of Desulfobulbia, BD2-11 terrestrial group, Kryptonia, and MB-A2-108 (Fig. 5). PCA axis 2 loadings indicated that increasing site scores corresponded to increases in relative abundances of Kapabacteria and Parcubacteria and decreases in relative abundances of Bacilli (Fig. 5).

**Fig 6:**
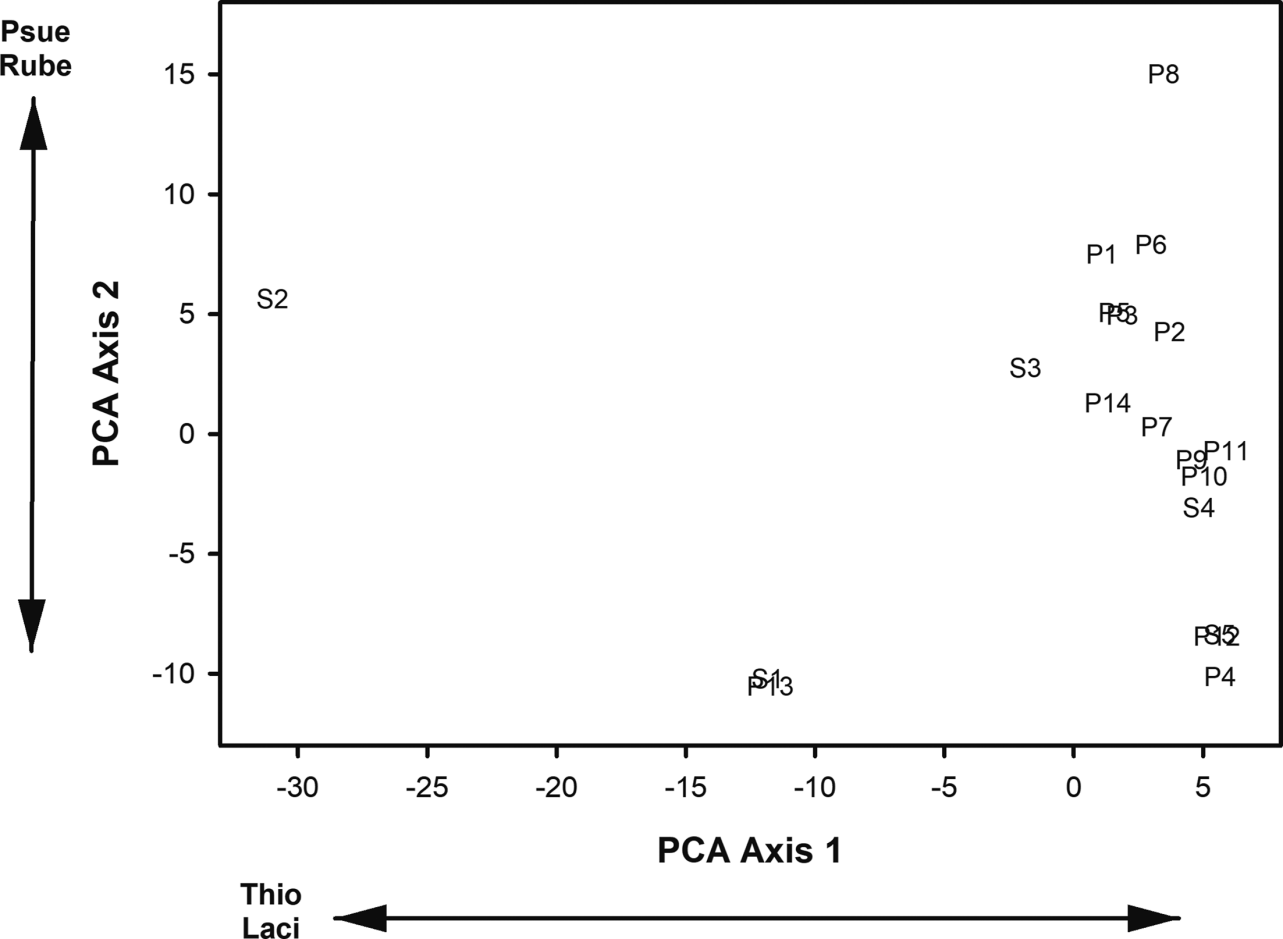
Site scores from the first two axes of the Principal Components Analysis of the relative abundances of microbial genera in fourteen supraglacial pools and five stream pools on the Hailuogou Glacier, Ganze Tibetan Autonomous Region, China, June 2018, and August 2019. Microbial genera abbreviations are: Thio – Thiobacillus; Laci – Lacihabitans; Pseu - Pseudanabaena PCC-7429; Rube – Rubellimicrobium. Supraglacial pool sites are those letter number combinations that begin with P and stream pool sites are those letter number combinations that begin with S.

None of the genera were found in all supraglacial pools and all stream pools. *Sphingomonas* was found in 92.8% (13 of 14) supraglacial pools and all stream pools. *Angustibacter*, *Flavobacterium*, and *Rhizorhapis* occurred in 92.8% of supraglacial pools and 80.0% (4 of 5) stream pools. Indicator genera of supraglacial pools were *KD3-10* (IV = 71.9, *P* = 0.031) and *Hymenobacteraceae* (IV = 70.2, *P* = 0.030). Indicator genera of stream pools were *Rhodopirellula* (IV = 79.4, *P* = 0.002), *Paludibacter* (IV = 60.0, *P* = 0.009), *Planctopirus* (IV = 60.0, *P* = 0.009), *Hydrogenophaga* (IV = 59.5, *P* = 0.017), *IMCC26207* (IV = 59.1, *P* = 0.009), *Lacihabitans* (IV = 54.4, *P* = 0.032), *Aquipuribacter* (IV = 51.8, *P* = 0.019), *BIyi10* (IV = 51.4, *P* = 0.035), *Staphylococcus* (IV = 51.2, *P* = 0.040)*, Hassallia* (IV = 50.2, *P* = 0.034), *Nitrosarchaeum* (IV = 49.0, *P* = 0.023), and *Sulfuricurvum* (IV = 45.8, *P* = 0.032). The first two axes from the genus PCA accounted for 32.3% of the variance in the data, and the eigenvalues from both axes were greater than the broken stick eigenvalues. PCA axis 1 loadings indicated that increasing site scores corresponded with decreasing relative abundances of *Thiobacillus* and *Lacihabitans* (Fig. 6). PCA axis 2 loadings indicated that increasing site scores corresponded to increases in relative abundances of *Pseudanabaena PCC-7429* and *Rubellimicrobium* (Fig. 6).

**Fig 7:**
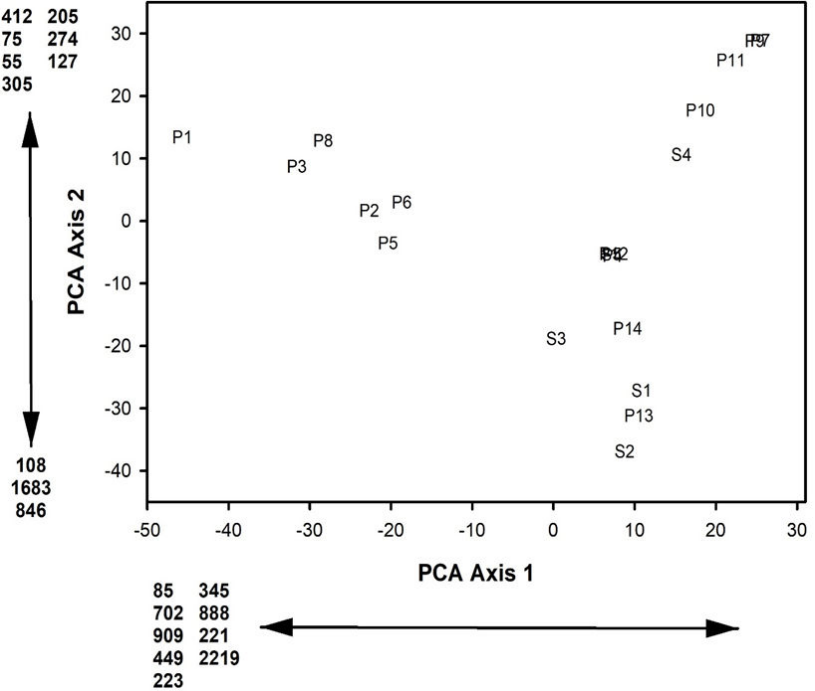
Site scores from the first two axes of the Principal Components Analysis of the relative abundances of microbial ASVs in fourteen supraglacial pools and five stream pools on the Hailuogou Glacier, Ganze Tibetan Autonomous Region, China, June 2018, and August 2019. Microbial ASV abbreviations are: 85 – ASV85; 702 – ASV702; 909 – ASV909; 449 – ASV449; 223 – ASV223; 345 – ASV345; 888 – ASV888; 221 – ASV221; 2219 – ASV2219; 412 – ASV412; 75 – ASV75; 55 – ASV55; 305 – ASV305; 205 – ASV205; 274 – ASV274; 127 – ASV127; 108 – ASV108; 1683 – ASV1683; 846 – ASV846. Supraglacial pool sites are those letter number combinations that begin with P and stream pool sites are those letter number combinations that begin with S.

None of the ASVs were found in all supraglacial pools and all stream pools. ASV 6 was found in 92.8% (13 of 14) of the supraglacial pools and 80% (4 of 5) of the stream pools. ASVs 1, 2, 5, 12, 13, 22, 26, 28, 30, 39, 43, 48, 49, 57, 59, 86, and 95 were also found in 85.7% (12 of 14) of the supraglacial pools and 80% of the stream pools. Supraglacial pools had 12 indicator ASVs and stream pools had 29 indicator ASVs (Table 5). The first two axes from the ASV PCA accounted for 27.7% of the variance in the data and the eigenvalues from both axes were greater than the broken stick eigenvalues. PCA axis 1 loadings indicated that increasing site scores corresponded with decreasing relative abundances of ASVs 85, 702, 909, 449, 223, 345, 888, 221, and 2219 (Fig. 7). PCA axis 2 loadings indicated that increasing site scores corresponded to increases in relative abundances of ASVs 412, 75, 55, 305, 205, 274, and 127 and decreases in relative abundances of ASVs 108, 1683, and 846 (Fig. 7).

**TABLE 5:** Summary of results from indicator species analysis conducted on ASVs from supraglacial pools and stream pools on the Hailuogou Glacier, Ganze Tibetan Autonomous Region, China, June 2018 and August 2019^*a*^

ASV #	Taxonomy	Importance value	*P* value
Supraglacial pools			
110	OLB14****	74.4	0.021
102	Terrimonas*	74.9	0.021
74	Ellin6067*	72.9	0.034
394	Fibrobacteraceae**	71.4	0.024
470	Rhodovastum*	71.4	0.027
97	Lineage IV***	68.6	0.049
369	Xanthobacteraceae**	68.2	0.042
400	Opitutus*	64.3	0.033
1290	0319–6G20***	64.3	0.035
560	AAP99*	64.3	0.038
334	Ferruginibacter*	64.3	0.039
858	Solirubrobacteraceae**	64.3	0.040
Stream pools			
319	Armatimonas*	71.2	0.015
142	Comamonadaceae**	67.5	0.049
446	Ellin6067*	64.8	0.020
407	Ferruginibacter*	62.1	0.020
1524	Ferruginibacter*	60.0	0.011
1146	Rhodoferax*	60.0	0.011
388	Rhizobacter*	60.0	0.011
1115	env.OPS 17**	60.0	0.011
359	Flavobacterium*	60.0	0.011
833	Pedomicrobium*	60.0	0.011
1268	Knoellia*	60.0	0.011
2364	Calothrix KVSF5*	60.0	0.011
1685	Unclassified*****	60.0	0.011
1183	Nitrosarchaeum*	60.0	0.011
3042	Rubellimicrobium**	60.0	0.011
485	Luteolibacter*	60.0	0.011
611	Prosthecobacter*	58.3	0.016
1055	Isosphaeraceae**	57.7	0.014
663	0319–6G20***	56.6	0.017
857	Sulfurifustis*	56.1	0.019
358	Methylotenera*	55.3	0.019
218	Lacihabitans*	55.6	0.034
762	Rhizobiales Incertae Sedis**	53.7	0.014
776	Hirschia*	53.9	0.039
384	KD4-96****	52.5	0.032
846	OLB12*	52.3	0.022
1683	Parablastomonas*	51.1	0.036
1320	Actimicrobium*	48.5	0.035
32	Sulfuricurvum*	44.7	0.036

^*a*^ Indicator ASVs at the lowest level of identification indicated with asterisks at the Genus*, Family**, Order***, Class****, and Phylum***** levels.

A distinct separation between supraglacial pools and stream pools did not occur within Fig. 5 to 7, which indicates that the microbial taxa composition at the class, genus, and ASV level did not differ between supraglacial pools and stream pools. MRPP results also confirmed that taxa composition at the class level (T = −0.030, A = 0.001; *P* = 0.422), genus level (T = 0.173, A = −0.002; *P* = 0.492) and ASV level (T = 0.446, A = −0.006; *P* = 0.610) did not differ between supraglacial pools and stream pools. The Mantel tests indicated that the greatest similarity in PCA axis 1 and 2 site scores occurred between the class and genus PCAs (Table 6).

**TABLE 6:** Summary of results from Mantel tests conducted to evaluate the similarity of Principal Components Analysis (PCA) axis 1 site scores and PCA axis 2 site scores among the PCAs conducted with the microbial class, genus, and ASV level from supraglacial pools and stream pools on the Hailuogou Glacier, Ganze Tibetan Autonomous Region, China, June 2018 and August 2019

Comparison	Mantel statistic	*P* value	% redundancy
Class PCA vs Genus PCA	0.623	< 0.001	38.9
Class PCA vs ASV PCA	0.391	0.007	15.3
Genus PCA vs ASV PCA	0.267	0.067	7.1

## DISCUSSION

### Supraglacial pool microbial community characteristics

Our results represent the first documentation of microbial taxa composition in supraglacial pool sediments on a debris-covered glacier in the monsoonal temperate region of Tibet. The relative abundance of the most abundant bacteria phyla (Proteobacteria, Actinobacteriota, Bacteroidota, Chloroflexi, and Cyanobacteria) was similar to cryoconite hole studies in the Antarctic and Arctic, where dominant phyla were Proteobacteria, Bacteroidota, Actinobacteria, and Cyanobacteria (4, 10). Proteobacteria in the Hailuogou Glacier supraglacial pools comprised 30% of ASVs compared with 18.7% in cryoconite holes of the Tibetan Plateau glaciers (16). The family Comamonadaceae, which constituted 13% of the ASVs in our samples, was comprised of 50% of the genus *Polaromonas. Polaromonas* is a well-known bacterial genus with cosmopolitan distribution inhabiting snow and ice habitats (11, 34, 42, 43). *Sphingomonas,* which comprised 8% of the Proteobacteria ASVs in the supraglacial pools, are key constituents of glacier ice (44) and can grow at low temperatures of 0-25°C (13). *Acidiphilium* were the third most abundant genus in the supraglacial pools and known to be active at temperatures < 5°C (45), which may explain their abundance within the supraglacial pools.

The relative abundance of Cyanobacteria in Hailuogou supraglacial pools was lower than the 9 to 40% Cyanobacteria relative abundance reported for a study of Antarctic and Arctic cryoconite holes (10) but were similar to the Cyanobacteria relative abundances reported in cryoconite holes on the Canada Glacier, Antarctica (18, 46). The Forni Glacier was sampled during multiple ablation seasons, and it was found that Cyanobacteria abundance varies annually and within the meltwater season (47). Interestingly, Cyanobacteria were undetected in 22 sites from supraglacial ponds and periglacial streams from a debris-covered glacier in Tibet (12). Notably, our supraglacial pools were intermediate in size between cryoconite holes and supraglacial ponds and exhibited turbidity and water depth conditions ranging from clear pools with shallow water depths and visible moss growth to highly turbid pools with deeper water depths. These factors, along with the known high sedimentation of monsoonal region glacier meltwaters during the ablation season (48, 49), and the habitat sampled (i.e., sediment versus water) may result in varying levels of Cyanobacteria relative abundance seasonally and annually at any glacier site.

### Best predictors of microbial response variables

We were surprised at the prominent role that Chironomidae larvae abundance played in influencing genera richness, indicator genera richness and *Polaromonas* relative abundance, which suggests that Chironomidae may exert a keystone species effect on microbial communities in supraglacial pools. In proglacial meltwater streams and supraglacial pools, predators of chironomids and other macroinvertebrates (i.e., fish) are absent and diatoms and algae dominate the food webs (50). Within the food webs, symbiotic relationships occur between the extracellular components of diatoms, algae (i.e., the phycosphere), and bacteria that could be important sources of nutrients for Chironomidae larvae (51). *Polaromonas* in Antarctica have been found to consume stress-induced carbohydrates released by diatoms before the carbohydrates could be oxidized (43). *Polaromonas vacuolata* bacteria surrounded by pigmented epibionts form phototrophic *Chlorochromatium aggregatum* consortiums (52), and in some lakes, bacterial consortiums have been found to constitute over 65% of the biomass (53). Diatoms and symbiont bacteria may serve as an important source of lipids for energy-intensive growth of chironomids in cold, oligotrophic environments (54–57). Bacteria have also been found to comprise 50% of the carbon requirements of chironomids in oligotrophic lakes compared with only 2% in hypereutrophic lakes (58). Moreover, chironomids have been known to selectively consume diatoms on algal surfaces due to their higher digestibility than lignin-rich filamentous algae (59), which could increase chironomid consumption of bacteria on diatom surfaces. Additionally, periphyton grazing by chironomids has been found to prevent the establishment of blue green algae in experimental stream channels and are thought to be responsible for the declines in spring diatom blooms in chalk streams (60, 61). Our results, previous studies, and the multiple feeding strategies of Chironomidae larvae (e.g., collector-gatherer, scraper, grazer) suggest that Chironomidae larvae serve as keystone species in supraglacial meltwater environments. We recommend future studies to examine the dynamics among bacteria, fungi, and invertebrates in supraglacial pools to understand the interactions among the taxa within these food webs.

The relationship of pH with *Angustibacter* and *Oryzihumus* relative abundance was similar to the findings that pH had a major influence in the number of bacterial OTUs and Shannon diversity index of cryoconite in cryoconite holes (15). pH also had a negative influence on alpha diversity in sediments of glacial fed rivers and lakes in southeastern Tibet (16). Studies of ancient ice cores have found *Sphingomonas* to be a prominent member of bacterial communities of ice (44). The relationship of increasing *Sphingomonas* abundance with glacier zone in our study could be due to the greater connectivity of lower altitude supraglacial pools with englacial conduit meltwater inputs, which would result in greater abundance of previously ice-entombed *Sphingomonas* cells.

The negative relationship we found with turbidity and *Acidiphilium* relative abundance may be occurring due to lower turbidity allowing enough sunlight to be received and used by photosynthesizing purple bacteria in low temperature (0-4^o^C) habitats. Additionally, supraglacial pools that contained the greatest relative abundance of *Acidiphilium* contained visible epilithic moss on the cobble-sized pool substrate which supports an environment where photosynthesis is taking place. The pool with the greatest relative abundance of *Acidiphilium* was located in the lower portion of the ablation zone and had the second greatest relative abundance of Chironomidae larvae. Although more is known about mesophilic, thermophilic, and extreme thermophilic species of *Acidiphilium* than cryotolerant *Acidiphilium*, the relationships between moss presence in the supraglacial pools and successional moss microbiomes, which have been found to change with deglaciation (62), would be a fruitful area to investigate. The relative abundance of *Acidiphilium* and Chironomidae larvae warrants further investigations into the function of this purple bacteria genus in relationship with moss and debris-covered glacier ecosystems.

### Taxa composition comparisons between supraglacial pools and stream pools

Although supraglacial pools and periglacial meltwater streams had unique indicator species at the class, genus, and ASV levels, taxa composition at the class, genus, and ASV taxonomic levels did not differ between supraglacial pools and stream pools of the Hailuogou Glacier Valley. There are three reasons for the similarity in taxa composition. First, the streams we sampled originated from the same historical icefield which has split into three valley glaciers within the past century (63). The historic connectedness is reflected in the similar microbial communities in the proglacial stream pools and supraglacial pools. The streams we sampled were mostly metakryal (T_max_ temp <2°C) and hypokryal sites (T_max_ temp <4°C) of the Hailuogou Glacier and Glacier number 3 that are heavily influenced by glacial meltwaters during the summer months. The Hailuogou sites had more similar temperatures between the supraglacial pools (1.2±0.38^o^C) and stream pools (4±1.56^o^C) than a study which examined supraglacial pond bacterial communities with periglacial stream communities in Linzhi, Tibet (12). In the Linzhi, Tibet study the periglacial stream sites were at least one km away from the glacier with greater water temperatures (>11.5°C) than the supraglacial ponds (<5°C) (12). Higher water temperatures indicate greater groundwater contribution or greater distance from the origin glacier which could be reflected by different microbial communities. Lastly, microbial taxa composition differs between the water column and benthic sediment. Sediment communities are depositional and are known to contain greater concentrations of DNA (4, 16), as sediment has had more time to develop and reflect cumulative historic conditions versus transient communities in flowing stream water. Additionally, freeze processes in lentic cryoconite hole habitats occur from the lid-down in Antarctica and could expunge bacteria from the water column and insert them into the sediments (4). Perhaps a similar process occurs in supraglacial pools.

In summary, the microbial community characteristics of supraglacial pools on the Hailuogou Glacier were similar to those in the Arctic and Antarctica at the phyla level but differences were detected in the relative abundance and variations occurred at the genus level. Our results highlight the impact that Chironomidae larvae had on the microbial communities. Subsequently, we recommend future research to examine Chironomidae, meiofauna, fungi, and bacteria throughout the melt season to elucidate interactions between this potential keystone species and other taxa in supraglacial pools. Our results comparing the stream and supraglacial pool sites suggest that historical glacier connectedness and torrential summer meltwater regimes may have long-term influence on the microbial communities in sediments even as glacier recession and separation occurs. Continued monitoring of glaciers and their ecosystems is critical for deciphering the impacts the melting cryosphere is having on the glacial and periglacial biological communities.

## Data Availability

Sequencing data have been deposited in the National Center for Biotechnology Information Sequence Read Archive with the BioProject Accession number PRJNA999376. All other data are available in the main text and supplementary files.

## SUPPLEMENTAL MATERIAL

Supplemental material spectrum.00754-24-s0001.docx Table S1.

ASM does not own the copyrights to Supplemental Material that may be linked to, or accessed through, an article. The authors have granted ASM a non-exclusive, world-wide license to publish the Supplemental Material files. Please contact the corresponding author directly for reuse.
