# Supplementary material for: Determinants of microbial community structure in supraglacial pool sediments of monsoonal Tibetan Plateau
Source: Microbiol Spectr. 2024 Jul 30;12(9):e00754-24. doi: 10.1128/spectrum.00754-24 (PMC11370254; doi:10.1128/spectrum.00754-24)
Supplement: Supplemental material — Table S1. [file spectrum.00754-24-s0001.docx]

**Supplemental Table 1** Summary of the difference in the small sample Akaike information criterion score (AICc) scores between each model and the model with the minimum AICc from the top ten models identified during stage 1 analyses of ten microbial response variables from supraglacial pools on the Hailuogou Glacier, Ganze Tibetan Autonomous Region, China, June 2018 and August 2019.

|  | **Gen Rich** | **AnguRA** | **OryzRA** | **AcidRA** | **SphiRA** | **PolaRA** | **IndgRA** | **IG Rich** | **PCA1** | **PCA2** |
| --- | --- | --- | --- | --- | --- | --- | --- | --- | --- | --- |
| meanarea | 21.71 | - | - | 8.69 | - | - | **-** | - | **-** | - |
| avgwaterdepth | - | 6.96 | - | - | - | - | - | - | - | - |
| avgsidepth | 19.35 | 6.75 | - | 9.30 | - | 8.38 | - | 5.87 | - | - |
| subrich | - | - | - | - | - | - | - | - | - | **0.00** |
| mngrsize | 21.51 | - | - | - | - | - | - | - | - | - |
| h20temp | 19.90 | - | 2.91 | 9.89 | 2.14 | 10.16 | 0.25 | 6.11 | - | - |
| pH | - | **0.00** | **0.00** | - | - | 10.17 | 1.18 | 7.12 | 0.93 | 1.65 |
| condspc | - | - | 3.35 | 6.46 | 2.22 | - | 2.62 | - | 2.44 | 1.65 |
| turb | 18.34 | 2.65 | - | **0.00** | 4.79 | 3.70 | - | 5.53 | 1.90 | 1.91 |
| difNB1SB1 | 21.05 | 1.70 | 2.08 | 5.70 | 5.04 | 8.40 | - | 7.86 | 1.71 | 1.17 |
| nbrpool5m | - | 6.11 | 2.40 | - | 5.37 | - | **0.00** | 7.77 | 2.83 | 2.10 |
| zone | - | - | 5.02 | 4.61 | **0.00** | 10.68 | 1.57 | - | - | 2.13 |
| elev | - | - | 3.57 | 6.79 | 0.81 | - | 0.95 | - | - | - |
| chirolarv | **0.00** | 1.30 | 1.52 | - | 5.49 | **0.00** | 2.84 | **0.00** | 1.96 | **-** |
| isotomidae | - | - | 4.72 | - | 4.60 | - | - | - | - | - |
| invert | 20.01 | 5.49 | - | 9.82 | - | 4.07 | 2.86 | 6.05 | 1.18 | 1.58 |
| pachirolarv | 16.39 | 6.58 | - | - | - | 8.24 | - | 7.77 | 1.72 | - |
| paisotomidae | - | - | 5.00 | - | 4.68 | - | - | - | 2.90 | - |
| painvert | 19.20 | 6.09 | - | 9.66 | - | 6.07 | **-** | 7.55 | **0.00** | 1.27 |

* Abbreviations for microbial response variables are: Gen Rich – genera richess; AnguRA – *Angustibacter* relative abundance; OryzRA – *Oryzihumus* relative abundance AcidRA – *Acidiphilium*, relative abundance SphiRA – *Sphingomonas* relative abundance PolaRA – *Polaromonas* relative abundance; IndgRA – indicator genera relative abundance; IG Rich – indicator genera richness; PCA1 – Principal components analysis axis 1 site scores; and PCA2 – Principal components analysis axis 2 site scores

**Abbreviations for predictor variables variables are: meanarea – mean pool surface area; avgwaterdepth – average water depth; avgsidep – mean ice surface to water surface depth; subrich – substrate richness; mngrsize – mean grain size; h20temp – water temperature; condspc – specific conductivity; turb – turbidity; difNB1SB1 – difference in distances of the pools from the northern and southern glacier boundaries; nbrpool5m – number of pools within 5 meters; zone – upper or lower region of the glacier; elev – elevation; chirolarv – Chironomidae larvae abundance; isotomidae – Isotomidae larvae abundance; invert – invertebrate abundance; pachirolarv – Chironomidae larvae occurrence; paisotomidae - Isotomidae larvae occurrence; painvert – invertebrate occurrence
